# A New Approach for Heterogeneity Corrections for Cs-137 Brachytherapy Sources

**Published:** 2015-06-01

**Authors:** S. Sina, R. Faghihi, A. S. Meigooni

**Affiliations:** 1 Radiation Research Center, School of mechanical engineering, Shiraz University, Shiraz, Iran; 2 Nuclear Engineering department, School of mechanical engineering, Shiraz University, Shiraz, Iran; 3 Comprehensive Cancer Center of Nevada, Las Vegas, Nevada, USA

**Keywords:** Brachytherapy, Heterogeneity correction, Treatment planning system

## Abstract

**Background:**

Most of the current brachytherapy treatment planning systems (TPS) use the TG-43U1 recommendations for dosimetry in water phantom, not considering the heterogeneity effects.

**Objective:**

The purpose of this study is developing a method for obtaining correction factors for heterogeneity for Cs-137 brachytherapy sources based on pre-calculated MC simulations and interpolation.

**Method:**

To simulate the effect of phantom heterogeneity on dose distribution around Cs-137 sources, spherical water phantoms were simulated in which there were spherical shells of bone with different thicknesses (0.2cm to 1.8cm with 0.1cm increment) at different distances (from 0.1cm to 10cm, with 0.5cm increment) from the source center. The spherical shells with 0.1cm thickness at different distances from 0.1cm to 10cm were used as tally cells. The doses at these cells were obtained by tally types F6, *F8, and *F4.The results indicate that the percentage differences between the doses in heterogeneity sections with the dose at the same positions inside the homogeneous water phantom vary when the distance of bone section from the source center increases, because of decreasing the average energy of photons reaching the bone layer. Finally, the results of Monte Carlo simulations were used as the input data of MATLAB software, and the percentage dose difference for each new configuration (i.e. different thickness of inhomogenity at different distances from the source) was estimated using the 2D interpolation of MATLAB.

**Results:**

According to the results, the algorithm used in this study, is capable of dose estimation with high accuracy.

**Conclusion:**

The developed method using the results of Monte Carlo simulations and the dose interpolation can be used in treatment planning systems for heterogeneity corrections.

## Introduction

The recommendations of the AAPM task group #43 (TG43, and TG43U1) has been widely used in most treatment planning systems for dosimetry of brachytherapy sources. One of the main defects of the TG-43 recommendations is considering the homogeneous water phantom, not taking the tissue inhomogenity into account. Variety of tissues (i.e. bone, soft tissue, air cavities, and etc) with different physical and radiological properties exists inside the human body. Accurate prediction of the dose in presence of heterogeneities is a very important issue in maximize the therapeutic benefit in brachytherapy and radiation therapy. The heterogeneity corrections in radiation therapy treatment planning have been the topic of different studies[1-5].

The purpose of this study is developing a new fast method for the heterogeneity corrections in brachytherapy treatment planning

## Material and Methods

### Monte Carlo Simulations

Today, the Monte Carlo codes have been accepted as the gold standard for medical dosimetry. MCNP4C Monte Carlo code has been widely used for dosimetry in brachytherapy[6, 7, 8]. In this study, MCNP4C code was used for heterogeneity correction for Cs-137 brachytherapy sources. This code is capable of simulating different photon and electron interactions. Various tallies of this code can be used for scoring the dose in the phantom.

In this study, first a spherical water phantom was simulated. The tally cells were spherical shells with thicknesses of 0.1cm. To score the dose at the tally cells, tally types F6, and *F8 (divided by the mass of the shell), and *F4 (multiplied by the mass absorption coefficient of the tissue) were used. Then the percentage differences between the results of these tallies were calculated.

In the next step, the spherical layers of bone with different thicknesses were simulated inside the phantom at different distances from the source. Finally the percentage difference between the dose in homogeneous and inhomogeneous phantom were obtained.

### Method for heterogeneity correction

In this step, the results of the simulations were used as the input data in the MATLAB software. The percentage dose difference for bone layers of other thicknesses in other distances were obtained using two dimensional interpolations in MATLAB for thick bone layers, and distance based correction factors for thin bone layers. 

## Results and Discussions

### Comparison of different tallies

**Figure 1:**
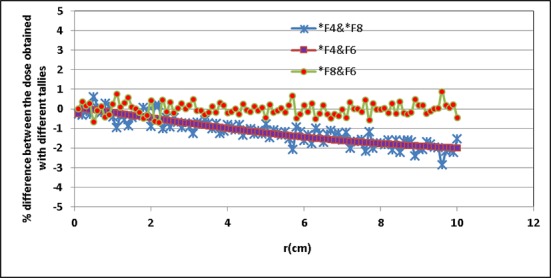
The percentage differences between the doses obtained by different tallies of MCNP4C.

The dose at different distances (0.1cm to 10cm) of water phantom were obtained using three common tally types *F4, F6, and *F8. Figure 1 shows the percentage differences between the results of the three tallies. According to the results, the percentage difference between the dose calculated by *F4 tally and the other two tallies, increase by increasing the tally cell distance from the source. The figure show that the maximum difference between *F4 tally and the other tallies was found to be 3%. While the difference between the doses obtained by tallies *F8 and F6 are less than 1% at all points. Therefore the tally F6 was used for obtaining the dose in heterogeneous phantoms containing bone layers.

### MCNP4c results for heterogeneous phantom

**Figure 2:**
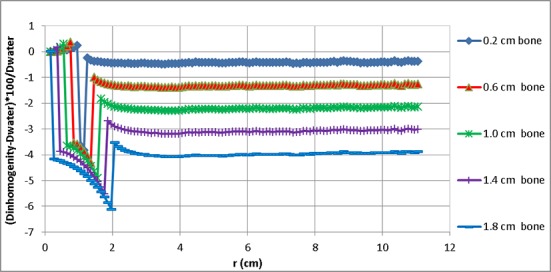
Percentage difference between the dose in inhomogeneous phantom and the dose in water phantom for different thicknesses of the bone layer (at r=1cm).

**Figure 3:**
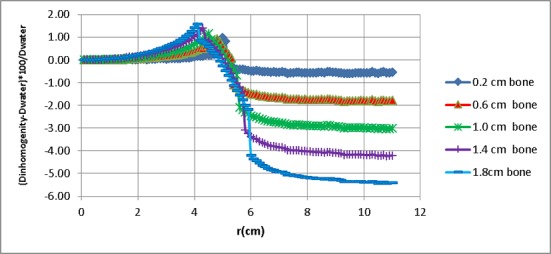
Percentage difference between the dose in inhomogeneous phantom and the dose in water phantom for different thicknesses of the bone layer at r=5cm.

**Figure 4:**
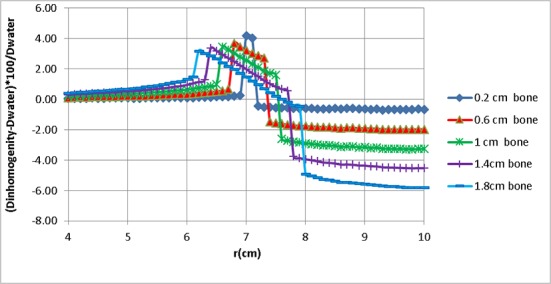
Percentage difference between the dose in inhomogeneous phantom and the dose in water phantom for different thicknesses of the bone layer at r=7cm.

The percentage differences between the doses in bone layers of heterogeneous phantom with different thicknesses (from 0.2 cm to 1.8 cm) located at 1 cm distance from the source center, and the doses at the same positions inside the homogeneous water phantom are shown in Figure 2. As it is obvious from the figure, the dose in the bone layer is less than the dose in water. This is due to the fact that in the photon energy spectrum at distance (r=1cm), the Compton interaction is more probable than photoelectric interaction; therefore the dose in the bone layer is less than the dose in water phantom. Figures 3 and 4 shows the dose variation inside the heterogeneity when the bone layers of different thicknesses are located at the distance of 5 and 7 cm from the source center.

**Figure 5:**
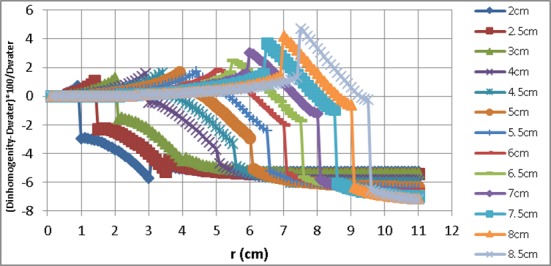
Percentage difference between the dose in inhomogeneous phantom and the dose in water phantom for bone thickness of 2cm located at different positions from the source center.

**Figure 6:**
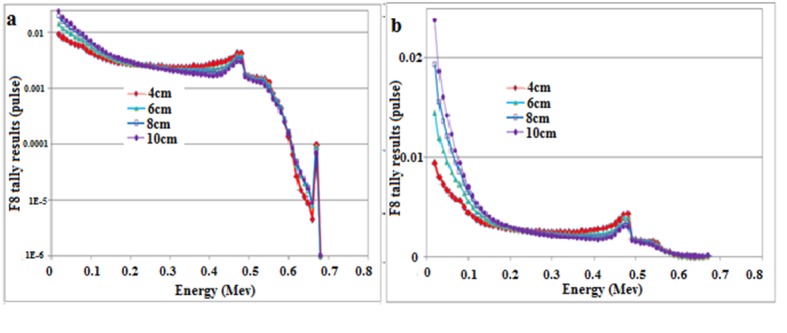
The spectrum of photons in different layers of phantom (a: logarithmic scale, b: linear scale)

Figure 5 shows the percentage dose difference between water phantom and the phantom containing bone layers with equal thicknesses, located at different distances from the source. The figure shows that the dose in bone layer increases by increasing the distance of the bone layer from the source center. This is due to the fact that increasing the distance of the bone layer from the source center has an important effect on energy spectrum of the source, and the average energy of the photons reaching the bone layer, decreases in bone layers far from the source. The photon energy spectrum at different distances from the source (obtained by tally F8) is shown in Figure 6. The average energy of photons reaching at each layer is obtained by equation 1.

$$E_{average}=\sum f_{i}E_{i}\;(1)$$

The results show that the average energies of photon reaching the far layers, decrease significantly, therefore photoelectric effect begins to become dominant, so that the dose in bone layer becomes more than the dose in water.

### Correction factor for thin inhomogenity

**Figure 7:**
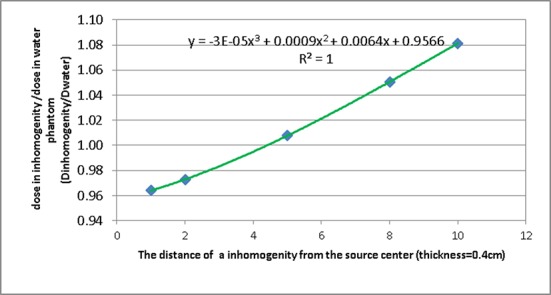
Dose ratio for thin bone layer.

The ratio of the dose in the thin inhomogenity layer (i.e. less than 0.4cm) to the dose at the same layer in homogeneous water phantom is shown in figure 7. According to the figure, a correction factor can be introduced for dose rate constant obtained in homogeneous water phantom according to equation 2.

$$\Lambda_{inhomogenity}=\Lambda_{water}\times \left(\frac{D_{inhomogenity}}{D_{water}}\right)$$

or

$$\Lambda_{inhomogenity}=\Lambda_{water}\times \left(-0.00003x^{3}+0.006x+0.956\right)\;(2)$$

Where *x* is the distance of the inhomogenity from the source center.

### The results of interpolation for thick inhomogenity

The MC simulated dose in inhomogeneous phantom with the dose in water phantom for a 2cm bone inhomogenity located at different distances was compared with those obtained by the new method (two dimensional interpolation and applying some correction factors for the points in the boundaries). The results indicate that the maximum error of the new method for dose estimation in heterogeneity layer is found to be less than 0.4%.

## Conclusion

A new method for inhomogenity correction for thick and thin layers of inhomogenities was proposed in this study. Different thicknesses of inhomogenities were simulated at different distances from the Cs-137 source using MCNP4C Monte Carlo code. The results of the simulations were inserted to a MATLAB program in matrix form. The 2D interpolation of MATLAB was used to predict the behaviour of the dose in bone heterogenities with different thicknesses, and in different distances from the source. The results indicate that the 2D interpolation can predict the changes in dose in presence of thick inhomogenities. For thin inhomogenities a distance dependent correction factor for dose rate constant have been proposed. 

The accurate results for bone inhomogenity shows that the new method can be generalised for other inhomogenities inside the phantom and other high energy brachytherapy sources. This method can be used in treatment planning systems for increasing the dosimetry accuracy. 
